# TumorAgDB1.0: tumor neoantigen database platform

**DOI:** 10.1093/database/baaf010

**Published:** 2025-02-13

**Authors:** Yan Shao, Yang Gao, Ling-Yu Wu, Shu-Guang Ge, Peng-Bo Wen

**Affiliations:** School of Medical Infand Engineering, Xuzhou Medical University, No. 209, Tongshan Road, Yunlong District, Xuzhou, Jiangsu 221004, China; Department of Histology and Embryology, Shantou University Medical College, No. 243, Daxue Road, Shantou, Guangdong 515063, China; School of Medical Infand Engineering, Xuzhou Medical University, No. 209, Tongshan Road, Yunlong District, Xuzhou, Jiangsu 221004, China; School of Medical Infand Engineering, Xuzhou Medical University, No. 209, Tongshan Road, Yunlong District, Xuzhou, Jiangsu 221004, China; School of Medical Infand Engineering, Xuzhou Medical University, No. 209, Tongshan Road, Yunlong District, Xuzhou, Jiangsu 221004, China

## Abstract

With the continuous advancements in cancer immunotherapy, neoantigen-based therapies have demonstrated remarkable clinical efficacy. However, accurately predicting the immunogenicity of neoantigens remains a significant challenge. This is mainly due to two core factors: the scarcity of high-quality neoantigen datasets and the limited prediction accuracy of existing immunogenicity prediction tools. This study addressed these issues through several key steps. First, it collected and organized immunogenic neoantigen peptide data from publicly available literature and neoantigen databases. Second, it analyzed the data to identify key features influencing neoantigen immunogenicity prediction. Finally, it integrated existing prediction tools to create TumorAgDB1.0, a comprehensive tumor neoantigen database. TumorAgDB1.0 offers a user-friendly platform. Users can efficiently search for neoantigen data using parameters like amino acid sequence and peptide length. The platform also offers detailed information on the characteristics of neoantigens and tools for predicting tumor neoantigen immunogenicity. Additionally, the database includes a data download function, allowing researchers to easily access high-quality data to support the development and improvement of neoantigen immunogenicity prediction tools. In summary, TumorAgDB1.0 is a powerful tool for neoantigen screening and validation in tumor immunotherapy. It offers strong support to researchers.

**Database URL**: https://tumoragdb.com.cn

## Introduction

With continuous advancements in cancer treatment technology, neoantigen-based immunotherapy has emerged as a promising therapeutic strategy, showing great clinical potential [1]. Such therapies activate the patient’s immune system to achieve a specific attack on tumor cells by leveraging tumor-specific neoantigens. Tumor neoantigens originate from somatic mutations in tumor cells. They are presented on the cell surface by major histocompatibility complex (MHC) molecules. T-cell receptors recognize these neoantigens, triggering an immune response [2]. Since neoantigens are not expressed in normal cells, they are not affected by immune tolerance mechanisms and possess high immunogenicity, making them ideal targets for tumor immunotherapy [3].

In recent years, the development of immune checkpoint inhibitors, tumor-infiltrating lymphocyte therapy, and neoantigen vaccines has significantly advanced personalized cancer treatment. For instance, immune checkpoint inhibitors targeting programmed cell death receptor 1 and cytotoxic T lymphocyte-associated protein 4 have achieved encouraging results in the treatment of melanoma and other cancers [4]. Nevertheless, the clinical success of immunotherapy remains dependent on the accurate identification and functional validation of tumor neoantigens. Techniques such as mass spectrometry (MS) and Enzyme - Linked Immunospot Assay are widely used to experimentally verify the immunogenicity of neoantigens. Recent studies show that neoantigen vaccines can reduce tumor recurrence in melanoma and glioblastoma. They achieve this by inducing T-cell responses. This finding has advanced research on tumor neoantigens [5].

Accurate prediction and identification of neoantigens are critical steps in personalized immunotherapy [6]. Neoantigen prediction workflows typically include whole-genome or exome sequencing, Human Leukocyte Antigen（HLA） typing, and affinity calculations for candidate neoantigens [7]. High-throughput omics and MS-based methods help predict neoantigens. Combined with bioinformatics algorithms, they effectively identify highly immunogenic neoantigens. Key steps in the prediction process include mutation identification, MHC binding affinity calculation, and immunogenicity assessment [8].

Currently, multiple neoantigen databases and tools have been developed, providing substantial data support for neoantigen prediction and screening. However, these databases still face limitations in practical application, restricting their broader use in personalized immunotherapy. For example, the DbPepNeo database lacks negative control data, affecting the accuracy of false positive rate evaluation [9]; the TANTIGEN database contains tumor T-cell antigens but lacks detailed information on experimental MHC-I binding and immunogenicity classification, limiting its utility for performance evaluation [10]; The TESLA consortium (Tumor - specific Epitope - based Systems for Life - science Analysis) is a collaborative platform for tumor neoantigen immunogenicity research where multiple groups share patients’ WES and RNA - seq data to screen and evaluate peptide immunogenicity. The TESLA database provides neoantigen data, but its validation of positive peptides is incomplete, and it lacks critical information such as wild-type sequences or gene names [11].

To address these limitations, we developed TumorAgDB1.0, a comprehensive database integrating experimentally validated tumor neoantigen data. TumorAgDB1.0 includes a collection of validated tumor antigen peptide datasets and simulated neoantigen data [12]. Due to the limited availability of clinically validated neoantigen immunogenicity data, our database incorporates immunogenic tumor neoantigen data validated in mouse models. This provides a valuable reference for further research. The Tools module in TumorAgDB1.0 provides 21 neoantigen features and 16 immunogenicity prediction metrics. It helps researchers understand factors influencing neoantigen immunogenicity. It also allows systematic tracking of advancements in neoantigen immunogenicity prediction tools. This will facilitate the development of more accurate immunogenicity prediction tools.

## Materials and methods

### Data collection

The primary data sources for TumorAgDB1.0 include published literature and publicly available tumor neoantigen-related databases. Specifically, we first conducted a systematic literature search using PubMed. Keywords such as “tumor,” “immunogenicity,” “neoantigen,” and “neoepitopes” were used. During the selection process, we carefully reviewed the abstracts and keywords of the articles. This allowed us to identify studies closely related to the immunogenicity of tumor neoantigens. We then performed an in-depth review of these selected articles to extract the relevant neoantigen data. In addition to literature searches, we also downloaded peptide binding and T-cell epitope datasets from the Immune Epitope Database (IEDB) database [13]. Neoantigen data were also retrieved from the National Cancer Institute (NCI) database. Furthermore, we incorporated data from the TESLA project, which provided 608 novel peptide sequences, further enriching the database.

All experimentally validated neoantigen data in the database come from literature and tumor neoantigen-related databases. In addition to literature data, we also searched and compiled experimentally validated neoantigen data from the IEDB database. Relevant experimental methods and cancer types were also collected. These data were cleaned, deduplicated, and integrated to form the experimentally validated dataset in our database. However, the amount of experimentally validated neoantigen data is still limited. It is insufficient to support the development of neoantigen prediction algorithms. To address this limitation, we further collected simulated peptide data. These peptides are mainly derived from published literature [14]. They were designed based on T-cell epitope information from IEDB. Their immunogenicity was evaluated by assessing T-cell activation and further refined through affinity calculations. This process allowed us to build an extended resource that includes a dataset of simulated peptides. This resource aims to provide more comprehensive data support for future research.

### Data annotations

For the neoantigen data we collected, we manually annotated each neoantigen. The annotation includes various aspects, such as peptide length and immunogenicity. We labeled peptides with immunogenicity as “1” and nonimmunogenic peptides as “0.” To determine whether a peptide has immunogenicity, we followed these criteria: if the peptide can induce a T-cell immune response, shows a positive result in experiments, or has an MHC binding affinity (IC50 value in nM) <500, we annotate the peptide as “1,” indicating that it has immunogenicity. Conversely, if the peptide cannot induce a T-cell immune response, shows a negative result in experiments, or has an MHC binding affinity (IC50 value in nM) >500, we annotate it as “0,” indicating that it lacks immunogenicity.

### Database implementation

The TumorAgDB1.0 website is built on open-source technology. The front end utilizes the Vue.js framework to enhance user interface responsiveness and interactivity. The backend uses Node.js to enable efficient server-side processing. MySQL serves as the primary tool for data storage and querying, supporting secure data storage and rapid retrieval. TumorAgDB1.0 is compatible with mainstream browsers and can be accessed on Google Chrome, Microsoft Edge, and Firefox. We have tested the database on these browsers to ensure compatibility and enhance user interaction.

## Results

### Database overview

**Figure 1.**
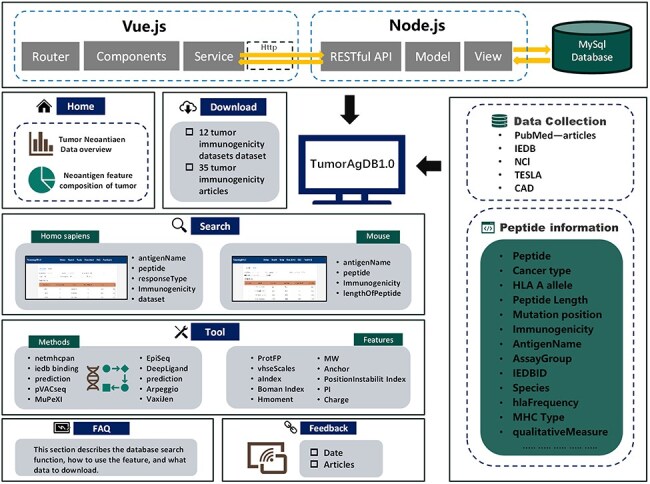
The top panel illustrates the overall architecture of TumorAgDB1.0, including the Vue.js frontend, Node.js backend, and MySQL database. The left panel presents the six functional modules of the database and the content included within each module. The right panel lists the data sources of the database, including IEDB, NCI, CAD, TESLA, and relevant research literature in the field, as well as various types of information about tumor neoantigens in the database, such as Peptide, Cancer type, HLA A allele, Peptide Length, Mutation position, Immunogenicity, AntigenName, AssayGroup, IEDBID, Species, hlaFrequency, MHC Type, and qualitativeMeasure. CAD is the cancer antigens database platform for cancer antigen algorithm development and information exploration.

TumorAgDB 1.0 provides an intuitive and user-friendly interface. It allows users to easily query and download tumor neoantigen data. The platform also supports neoantigen feature calculation and offers tools for predicting neoantigen immunogenicity. The database architecture is shown in Fig. 1. It consists of six core modules: (I) Home, (II) Search, (III) Tools, (IV) Download, (V) FAQ, and (VI) Feedback. The Home page provides a brief introduction to TumorAgDB 1.0 and its key features, helping users quickly understand the platform. The FAQ page explains the database functions and usage steps in detail. It helps users get started and utilize the platform efficiently. At the bottom of the FAQ page, we have compiled 35 high-impact research articles on tumor neoantigen immunogenicity prediction. Users can browse and download these resources as needed.

If users encounter any issues or have feedback, they can contact us via the email provided on the Feedback page. We are committed to providing prompt and professional support to ensure the platform meets the needs of tumor immunology research effectively.

### Data query

TumorAgDB1.0 contains two types of data: human neoantigen data and mouse neoantigen data. On the search interface, we divide the search options into two main modules based on species: “Homo sapiens” and “Mouse.” This classification helps users quickly locate relevant datasets according to their research needs. In the human neoantigen search module, the database provides multiple search options. Users can search based on five criteria: antigenName, peptide, responseType, dataset, and immunogenicity. For more complex queries, we offer an advanced search feature. This feature allows users to combine multiple search criteria and input several parameters to query human neoantigen data simultaneously. This flexible search capability helps users precisely locate specific neoantigen data. With advanced search, users can retrieve detailed information about neoantigens under specific conditions, covering 41 different attributes. Details about the search criteria and the retrieved data can be found in Supplementary Table S1. For example, users can filter neoantigens by setting the immunogenicity value to “1” or “0.” This helps identify immunogenic or nonimmunogenic neoantigens and their associated data. This functionality helps researchers quickly identify and analyze neoantigens related to immunogenicity. If users need to query immunogenicity data from different datasets, they can combine the dataset and immunogenicity criteria in the advanced search to refine their results further.

Currently, experimentally validated human neoantigen data are relatively limited. To address this gap, our database also includes tumor neoantigen data cultivated and validated in mice. When human data are insufficient, these mouse neoantigen data serve as valuable reference resources for researchers. They can be used for comparative analysis or to expand datasets for neoantigen studies. Specific search criteria and details of the mouse neoantigen data can also be found in Supplementary Table S1.

### Features affecting neoantigen immunogenicity

This database integrates multidimensional features of tumor neoantigens, including the physicochemical and immunological properties of amino acid sequences [15]. Specifically, physicochemical properties cover molecular weight, isoelectric point, hydrophobicity, polarity, and molecular volume. Studies using cryo-electron microscopy and X-ray crystallography reveal the importance of hydrophobic interactions in neoantigen recognition. These findings suggest that hydrophobic residues greatly influence the specific recognition of mutated peptides [16]. These physicochemical properties provide critical insights into the structural stability of peptides, aiding in the assessment of their binding affinity to MHC molecules.

In addition, the database includes essential immunological features, such as TAP transporter scores, mutant peptide abundance, entropy, and anchor positions [17]. These features are derived from tumor neoantigen literature. They reflect the efficiency of peptides in antigen processing and presentation. This indicates their potential to trigger immune responses.

The database combines physicochemical, sequence diversity, structural, biological, genetic, and antigen-processing characteristics and provides calculation methods for 21 neoantigen features. These features directly impact neoantigen immunogenicity and offer data support for developing immunogenicity prediction tools. The database enables multi-level analysis of neoantigen immunogenicity: a description and statistical overview of these features are provided in Supplementary Table S2. All features and their calculation methods are available on our website (https://tumoragdb.com.cn/#/tool-feature).

### Collection of tumor immunogenicity prediction tools

Neoantigens are peptide fragments generated by mutations in tumor cells, distinct from those in normal cells. These peptides can be recognized by the immune system, triggering an antitumor immune response. To accurately predict which peptides can be recognized as neoantigens, two main methods are used: peptide-MHC binding affinity prediction and artificial intelligence (AI)-based immunogenicity prediction. TumorAgDB1.0 integrates 16 immunogenicity prediction pipelines, covering various algorithms, platforms, and databases (see Supplementary Table S3 for details). These pipelines are divided into two categories: one based on peptide-MHC binding affinity and the other based on AI algorithms for immunogenicity prediction.

First, the database includes several tools designed to assess peptide-MHC binding affinity. These tools extract protein sequences from tumor samples to identify potential mutation regions. Mutated regions often contain tumor-specific peptide fragments, which could become neoantigens. Computational methods are then used to predict the binding affinity between peptides and different MHC molecules. Peptides with high binding affinity are more likely to be effectively presented and recognized by the immune system. Next, these high-affinity peptides undergo immunogenicity screening to evaluate whether they can activate T-cell responses. This often requires integrating additional factors, such as T-cell receptor recognition and immune evasion mechanisms. Tools included in the database, such as NetMHC [18], IEDB Analysis Resource—MHC I Binding Predictions, and MHCPred [19], are mature and reliable. However, they may have limitations, especially in predicting MHC-II molecules and considering the complex tumor microenvironment.

Additionally, the database integrates AI-based neoantigen prediction tools. These tools train AI models using large amounts of immunological data. The AI models use peptide sequence information, mutation types, and amino acid physicochemical properties as input features to predict which peptides are likely to be immunogenic. Common AI methods, including deep learning and traditional machine learning, help identify potential immunogenic peptides. After training, the AI models can predict the immunogenicity of new peptides and assess whether they can induce T-cell immune responses. Tools like NeoPredPipe [20], pVACtools [21], DeepNeo [22], and MuPeXI [23] are included in the database. These tools handle large and complex biological data, offering strong predictive capabilities, particularly when integrating various immunogenicity factors. However, the accuracy of AI methods depends on the quality of the training data, and the “black-box” nature of the models makes the prediction process less interpretable.

By integrating these two approaches, TumorAgDB1.0 provides researchers with comprehensive immunogenicity prediction tools. It aids in the accurate analysis of neoantigen data and supports clinical applications in tumor immunology.

### Neoantigen data statistics and downloads

**Figure 2.**
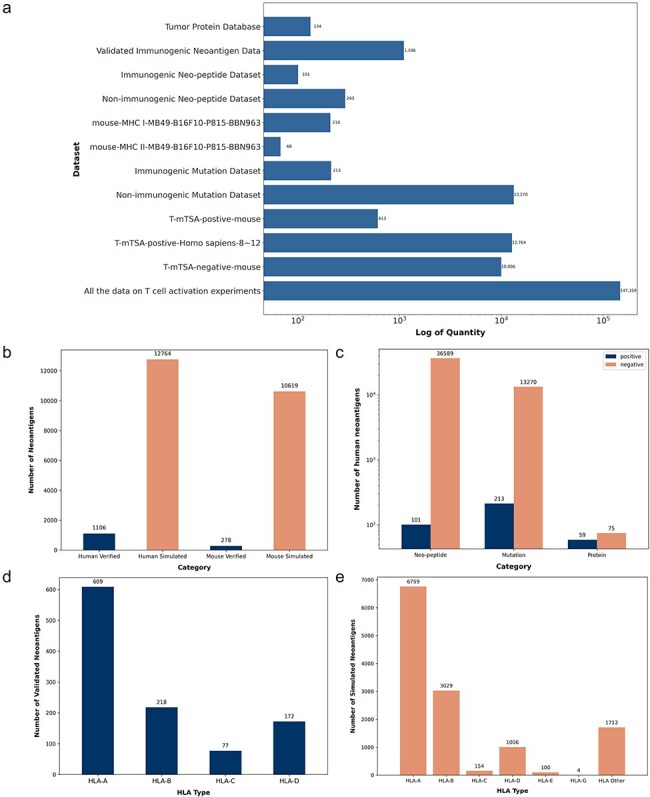
Neoantigen data statistics. (a) Distribution of neoantigen counts across 12 categorized datasets on the download interface. (b) Counts of validated and simulated neoantigens in human and mouse datasets. (c) Counts of immunogenic and nonimmunogenic neoantigens categorized by peptide length, including short peptides, mutated peptides, and tumor proteins. (d) Four HLA alleles matched with validated human neoantigens. (e) Six HLA alleles matched with simulated human neoantigens.

We conducted a systematic statistical analysis of the collected neoantigen data. TumorAgDB1.0 includes neoantigens from 15 different cancer types or cell lines. These cancer types include colon adenocarcinoma, skin cutaneous melanoma, rectum adenocarcinoma, breast invasive carcinoma, esophageal carcinoma, cervical cancer, cholangiocarcinoma, pancreatic adenoma and adenocarcinoma, lung adenocarcinoma, kidney renal clear cell carcinoma, stomach adenocarcinoma, sarcoma, uterine corpus endometrial carcinoma, bladder cancer, and mast cell leukemia. The collected neoantigen data have been organized into 12 categorized datasets. The number of neoantigens in each dataset is shown in Fig. 2a. All datasets are available for download on our official website (https://tumoragdb.com.cn/#/download). Given the limited availability of experimentally validated human neoantigens, we supplemented the database with murine tumor neoantigen data. As a result, TumorAgDB1.0 provides neoantigen data from two species. The database includes 1106 experimentally validated neoantigens. Figure 2b shows that we collected a larger number of simulated datasets. These simulated datasets can complement validated data to support the development of immunogenicity prediction tools and reduce model overfitting caused by data scarcity. Additionally, we analyzed the distribution of neoantigens by peptide length. This includes tumor proteins longer than 25 amino acids, mutated peptides of 20–25 amino acids, and short peptides of 8–12 amino acids (Fig. 2c). The analysis shows that neoantigens of 8–12 amino acids are the most abundant. We also examined the association of validated human neoantigens with four common HLA alleles (Fig. 2d). Among these, HLA-A had the highest frequency and the largest proportion. For simulated data, we mapped neoantigens to six common HLA alleles (Fig. 2e). Similarly, HLA-A was the most represented allele.

In conclusion, TumorAgDB1.0 provides a comprehensive and multidimensional neoantigen dataset. It covers a wide range of cancer types and detailed immunological characteristics. This database offers valuable support for neoantigen research and immunogenicity prediction tool development and contributes to advancing cancer immunotherapy research.

## Discussion

The TumorAgDB1.0 developed in this study provides a comprehensive database platform for tumor neoantigen research. It aims to offer richer and more reliable data, features, and algorithm information to support the development and optimization of tumor neoantigen immunogenicity prediction tools. This will help advance neoantigen screening in cancer immunotherapy. Compared to existing databases, TumorAgDB1.0 integrates several experimentally validated tumor neoantigen datasets and also includes simulated tumor-specific neoantigen data generated through affinity calculations. These additional datasets greatly expand the data available to researchers and provide more in-depth analysis support for neoantigen identification.

One key feature of TumorAgDB1.0 is its integration of various tumor neoantigen characteristics. It includes 21 critical features, such as sequence diversity, physicochemical properties, structural features, biological properties, genetic traits, and antigen processing characteristics. These features are essential for immunogenicity prediction. The platform offers rich feature information, which strongly supports the development of neoantigen prediction tools and lays a solid foundation for optimizing immunogenicity prediction algorithms. Furthermore, TumorAgDB1.0 integrates various immunogenicity prediction tools based on machine learning [24] and deep learning algorithms [25]. This allows researchers to conveniently compare and select different algorithms on a unified platform, exploring and validating innovative prediction methods.

Although TumorAgDB1.0 has made significant progress in data and feature integration as well as tool incorporation, it still faces some limitations. The primary issue is the data volume. While the database integrates multiple validated tumor neoantigen datasets and provides simulated neoantigen data, the overall amount of data remains relatively limited. In particular, experimentally validated neoantigen data, due to the limited number of available studies and data sources, do not cover all tumor types and immune responses. This may hinder further improvement in the accuracy of neoantigen prediction. As research continues, more experimental data may be included to enhance the comprehensiveness and diversity of the database. Additionally, the platform currently focuses primarily on providing feature information and calculation methods, but there is room for improvement in areas such as the intuitive presentation of specific feature calculations. Although the platform integrates several immunogenicity prediction tools, it does not yet include all mainstream algorithms. This includes some emerging deep learning methods. As technology progresses, adding more algorithms will help improve prediction accuracy and diversity.

To address these limitations, future updates to TumorAgDB1.0 will display specific calculation values for each feature. We will also integrate the tumor neoantigen immunogenicity prediction tools developed by our team. This will further enhance the platform’s usability and functionality. At the same time, by continuously accumulating experimental validation data and introducing more advanced prediction algorithms, TumorAgDB1.0 will strengthen its prediction capabilities and accuracy. Ultimately, it aims to become a more comprehensive and powerful platform in the field of neoantigen prediction and cancer immunotherapy research. In conclusion, TumorAgDB1.0 aims to be a continuously updated, multidimensional resource platform. It provides essential data support and serves as a reference for advancing cancer immunotherapy and optimizing personalized treatment strategies.

## Supplementary Material

baaf010_Supp

## Data Availability

All data and resources in TumorAgDB1.0 are freely accessible at https://tumoragdb.com.cn.
